# Knowledge Translation for Improving the Care of Deinstitutionalized People With Severe Mental Illness in Health Policy

**DOI:** 10.3389/fphar.2019.01470

**Published:** 2020-01-21

**Authors:** Izabela Fulone, Jorge Otavio Maia Barreto, Silvio Barberato-Filho, Marcel Henrique de Carvalho, Luciane Cruz Lopes

**Affiliations:** ^1^Pharmaceutical Sciences Graduate Course, University of Sorocaba, UNISO, Sorocaba, Brazil; ^2^Fiocruz School of Government, Fiocruz Brasília, Oswaldo Cruz Foundation, Brasília, Brazil; ^3^Veredas Institute, Brasília, Brazil

**Keywords:** evidence-informed policy, knowledge translation, health policy, policy-making, deinstitutionalization, mental health

## Abstract

**Background:**

Knowledge translation (KT) is an effective strategy that uses the best available research evidence to bring stakeholders together to develop solutions and improve public health policy-making. Despite progress, the process of deinstitutionalization in Brazil is still undergoing consolidation, and the changes and challenges that are involved in this process are complex and necessitate evidence-informed decision-making. Accordingly, this study used KT tools to support efforts that aim to improve the care that is available to deinstitutionalized people with severe mental disorders in Brazil.

**Methods:**

We used the Supporting Policy Relevant Reviews and Trials tools for evidence-informed health policymaking and followed eight steps: 1) capacity building; 2) identification of a priority policy issue within a Brazilian public health system; 3) meetings with policy-makers, researchers and stakeholders; 4) development of an evidence brief (EB) that addresses the problem of deinstitutionalization; 5) facilitating policy dialogue (PD); 6) the evaluation of the EB and PD; 7) post-dialogue mini-interviews; and 8) dissemination of the findings.

**Results:**

Capacity building and meetings with key informants promoted awareness about the gap between research and practice. Local findings were used to define the problem and develop the EB. Twenty-four individuals (policy-makers, stakeholders, researchers, representatives of the civil society, and public defense) participated in the PD. They received the EB to subsidise their deliberations during the PD, which in turn were used to validate and improve the EB. The PD achieved the objective of promoting an exhaustive discussion about the problem and proposed options and improved communication and interaction among those who are involved in mental health care. The features of both the EB and PD were considered to be favorable and helpful.

**Conclusions:**

The KT strategy helped participants understand different perspectives and values, the interpersonal tensions that exist among those who are involved in the field of mental health, and the strategies that can bridge the gap between research and policy-making. The present findings suggest that PDs can influence practice by promoting greater engagement among stakeholders who formulate or revise mental health policies.

## Background

Knowledge translation (KT) is a dynamic and interactive process that uses evidence to make decisions and take actions that can improve health outcomes and reduce health inequities, particularly in low- and middle-income countries (LMICs) (Boyko et al., 2012).

Overall, there are different complexities and barriers that impede the application of KT for public health action in LMICs: deficits in knowledge production, the application of the available knowledge, and the use of strategies that are based on the best available evidence (Malla et al., 2018). When resources are scarce and there are strong sociocultural interferences, the translation and dissemination of knowledge can be adversely affected by contextual and local limiting factors (Newlin and Webber, 2015).

In order to promote the appropriate use of scientific evidence in the development and implementation of public health policies, KT platforms such as the Evidence-informed Policy Network (EVIPNet), which is supported by the World Health Organization (WHO), have been established to support health policy-making in Africa, Asia, and the Americas (Moat et al., 2014). The main objective of the EVIPNet is to facilitate the use of scientific knowledge in the formulation and implementation of health policies. Specifically, it focuses on the preparation of evidence briefs and policy dialogues, and adopts an approach that is similar to the Supporting Policy Relevant Reviews and Trials (SUPPORT) method (Moat and Lavis, 2014).

KT platforms are change agents that have a positive impact on policy decisions, interest group interactions, and health systems (Ongolo-Zogo et al., 2018). The use of KT platforms in Uganda, Cameroon, and Lebanon demonstrate the positive impact of such platforms: the promotion of awareness, acceptance, and adoption of research-based knowledge, achievement of the health goals, reallocation of resources, and identification of the sources of conflicts (Yehia and El Jardali, 2015; Ongolo-Zogo et al., 2018).

Evidence briefs should rely on the best available systematic reviews to delineate the important aspects of the issue in question. It must integrate global evidences and local knowledge to inform deliberations about health policies among policy-makers and stakeholders (Lavis et al., 2009a). Policy dialogues use the evidence brief as primary input to subsidise the deliberations followed by the views, experiences and tacit knowledge of different actors, who will be affect or involved by future decisions (Lavis et al., 2009b; El-Jardali et al., 2014; Yehia and El Jardali, 2015).

Since its inception in Brazil in 2007, EVIPINet has been focusing on promoting the use of scientific knowledge in the decision-making processes of the Brazilian Health System, the development of innovative strategies in health management, and the facilitation of technical cooperation regarding KT among the participant countries (Evipnet-Brazil, 2019). The Brazilian network consists of the representatives of different institutions and subject-matter experts (Dias et al., 2014).

Accordingly, in response to the need for and challenges in the promotion of evidence-informed health policy-making in the largest city in the state of São Paulo (Sorocaba), a working group was constituted at the University of Sorocaba in 2016. This team, which consisted of researchers, doctoral students, and health professionals, was denominated as *Seriema* (Evidence Services for Monitoring & Evaluation in Health Policy).

The Seriema group aims to suggest and contribute to health initiatives and formulate evidence-based public policies. This group works collaboratively with the Health Department of Sorocaba, which oversees 48 additional cities in São Paulo that are together inhabited by more than three million individuals (Brazil, 2018).

This group seeks to design research studies in accordance with the needs of Brazilian policy-makers specially supporting deinstitutionalization in Brazil (mainly in region of Sorocaba).

### Mental Health in the Region of Sorocaba

In the 1980s, the history of Brazilian mental health was marked by serious denunciations of mistreatment, lack of hygiene and care for patients with mental disorders who lived in psychiatric hospitals, mainly in the region of Sorocaba (São Paulo), Rio de Janeiro (Rio de Janeiro), and Barbacena (Minas Gerais) (Vidal et al., 2008; Emerich and Yasui, 2016). Social and political mobilizations that advocated for psychiatric reform and the approval of Federal law no. 10216 in 2001 accelerated the process of deinstitutionalization. It also led to the understanding that hospitalization must be the last treatment option for patients with mental disorders. Consequently, the right to receive community care services was promulgated (Brazil, 2001; Silva and Rosa, 2014).

Sorocaba has a population of approximately 671,186 inhabitants and a high human development index (0.8), and its economy is based on industries and commerce (Brazil, 2019). The city has an adequate health-care infrastructure, and its hospitals provide services to the (almost three million) inhabitants of the tertiary care level of 48 municipalities in southwest São Paulo (Brazil, 2018). These municipalities are smaller than Sorocaba, their economies are diversified, and their high human development index ranges from 0.6 to 0.8 (Brazil, 2019). Mental health care services are not available in all 48 municipalities. Therefore, these municipalities belong to a network of mental health care institutions that are connected at the primary, secondary, and tertiary level (Brazil, 2019).

The Sorocaba region housed the largest mental asylum in the country (i.e. high number of psychiatric beds) (Cayres, 2015). The seven asylums in this region were among the ten largest Brazilian asylums that had the highest mortality rate between 2004 and 2011. Most of these deaths were due to an unknown cause, and they were especially common during the colder months of the year; the age of the youngest patient who died under these circumstances was approximately 53 years (Garcia, 2012; Cayres, 2015). In addition, there was a high number of resident patients who did not have the requisite civil documentation, and the number of mental health professionals was less than half of the number that was specified by the federal legislation (Garcia, 2012; Emerich and Yasui, 2016).

During the second half of the 1990s, there were 72,514 psychiatric beds in the Brazilian public health sector. In Brazil, the number of beds had reduced to 52,962 in 2001; in 2014, there were 25,988 psychiatric beds across the 167 psychiatric hospitals that were located in the 116 municipalities of the 23 states (Brazil, 2005; Brazil, 2015). In 2014, the Psychosocial Census of the State of São Paulo identified 53 psychiatric hospitals across 39 municipalities, seven of which were located within the Sorocaba region and together housed 2,273 patients (Cayres, 2015).

On the basis of the aforementioned census data, the federal, state, and municipal bodies signed an agreement that they would ensure the gradual deinstitutionalization of patients with mental disorders and the closure of the seven asylums in the region (Brazil, 2012). However, the deinstitutionalization process did not proceed in the same manner across the different regions of Brazil. Specifically, in regions where the number of patients that were admitted to the hospitals was very high, the institutions were underequipped to provide ambulatories and community services. This demonstrated the insufficiency and fragility of the services that were available to meet the demands of the patients (Vidal et al., 2008). However, a few community mental health care services (e.g. Psychosocial Care Center, Therapeutic Residential Service, and the Back Home Federal Program) have been found to be effective (Brazil, 2015). Nevertheless, some of the key principles that have been recommended by the WHO are not adhered to, primarily due to the following reasons: insufficient funding, qualitative and quantitative human resource deficiency, poor infrastructure, a lack of political resources and intensive follow-up care, the absence of an integration between services and fragile social mobilization (WHO, 2014; Brazil, 2015).

In October 2016, the Seriema organised the first workshop on evidence-based health policy during which the deinstitutionalization of patients with mental disorders was ascribed the highest priority among all other health policy-related issues. Subsequently, the State Health Department of the Sorocaba region contacted the Seriema group with the objective of signing a partnership and helping them formulate public policies that are related to deinstitutionalization. This represented an important opportunity to subsidise the policy and collaborate with the State Health Department. This allowed them to adapt their actions and strategies to improve the care of deinstitutionalized individuals with mental disorders in Sorocaba and the neighboring regions.

Since the use of KT is one of the challenges that is currently faced by the health systems in LMICs, the present study investigated the means by which the care of deinstitutionalized individuals with severe mental disorders can be enhanced using KT tools.

## Methods

**Figure 1:**
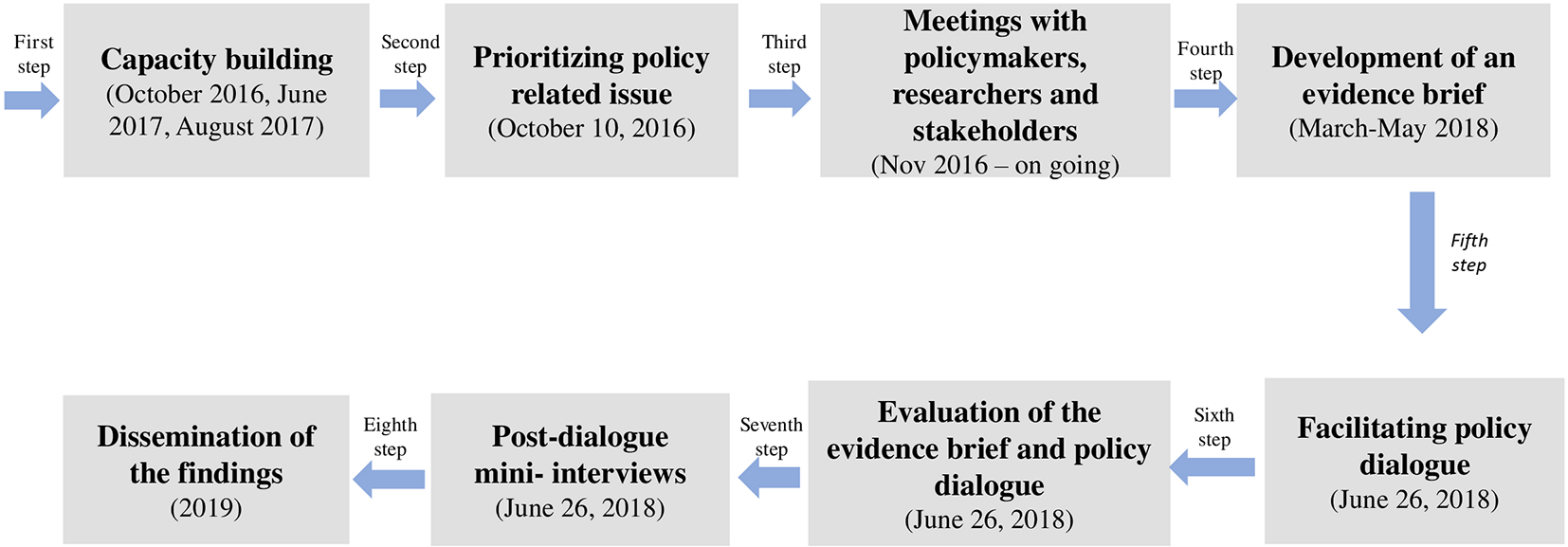
Eight steps used on KT*. *Adapted from Yehia; El Jardali (Yehia and El Jardali, 2015).

We used the SUPPORT Tools (Lavis et al., 2009a; Lavis et al., 2009b) for evidence-informed health Policymaking, which includes the following eight steps (**Figure 1**) for KT:

1. Capacity building
  There was a need to conduct capacity building workshops that addressed evidence-informed policy-making and provided technical training on the use of SUPPORT tools for relevant stakeholders. Therefore, in 2016 and 2017, three workshops were conducted to provide training and raise awareness. In addition, there was the possibility of addressing topics of interest.
2. Prioritizing and supporting evidence-informed policy-making
  The first step was to prioritise policy-related issues. The Seriema group provided a set of criteria that were to be used to select important topics, and it included questions about public perceptions and the impact of the problem (see **Supplementary Materials—Table S1**).
  In the first workshop, 40 participants fulfilled the criteria and they discussed their most pressing issues. Deinstitutionalization was identified as the most important health policy-related issue by the workshop participants. With regard to the means by which the care of deinstitutionalized people with severe mental disorders can be improved, the participants underscored the need for further evidence and to address policy-related challenges at both the national and regional levels. The chronology of events that have led to the current state of the mental health care systems in Brazil can be summarised as follows: (i) asylums provided inadequate services to their patients with mental disorders; (ii) there was immense pressure to shut down the seven psychiatric hospitals in the region; and (iii) important changes have been made to Brazilian mental health policies.
3. Meetings with policy-makers researchers and stakeholders
  A number of meetings were organised with policy-makers and stakeholders to clarify and define the problem, gather information about the status quo that could promote dialogue, and identify other key informants who could provide further insights.
4. The development of a policy brief that addresses the problem of deinstitutionalization
  Once the issue of deinstitutionalization was prioritised, the focus was geared towards gathering a wide range of evidence on the various aspects of the issue. Therefore, a systematic review of literature was undertaken. First, a well-defined search strategy was used to retrieve relevant research articles from research databases. The search focused elements for policies that were related to the care of deinstitutionalized patients with mental disorders (see **Supplementary Materials Data Sheet 1**).
  Between March and May 2018, we prepared a policy brief, which defined the problem and five evidence-based options to address the issue of deinstitutionalization. The evidence was contextualized to the Brazilian scenario, based on the recommendations of the policy-makers, subject-matter experts, and experts in the field of mental health.
5. Facilitating policy dialogue
  The policy brief was circulated to the participants 30 days prior to the dialogue to inform them of the deliberations of the meeting. A group of 24 individuals, which entailed an equal representation of policy-makers, health-care providers, researchers, and representatives of the community and public defense sectors, participated in the policy dialogue (see **Table 1**).
  The dialogue was conducted in accordance with the method that has been described by the SUPPORT tools and Chatham House rules. It was intended to achieve the following: participant commitment and transparency, an appropriate duration of dialogue, adequate group size and representation of the participants, skilful facilitation of problem-focused discussions (i.e. five options to address the policy issue), equity, key implementation considerations, and role distribution.
6. The evaluation of the evidence brief and policy dialogue
  The evaluation of the evidence brief and the policy dialogue was based on an adapted version of Lavis (2009) (Lavis et al., 2009a; Lavis et al., 2009b). Specifically, two surveys were administered to the participants (i.e. prior to dialogue and during the dialogue for those who did not complete it the first time). It consisted of items that required the respondent to assess the evidence brief and indicate the extent to which the policy dialogue was helpful on a rating scale that ranged from 1 (very unhelpful) to 7 (very helpful).
7. Post-dialogue mini-interviews
  During the policy dialogue, the stakeholders were invited to participate in a video-recorded interview. In this interview, they were required to describe the insights that they gained from the dialogue. For this purpose, we posed the following two questions: a) How did the policy dialogue change your perspective about the problem in question? and b) What actions should be taken to address the problem in question?
8. Dissemination of the findings
  The evidence brief was uploaded to the EVIPNet-Brazil secretariat webpage (http://brasil.evipnet.org/), where it is currently available for free download by all who are interested. A summary of the evidence brief and the policy dialogue will also be made available. Further, the federal government will order 100 prints of the evidence brief.

**Table 1:** A profile of the stakeholders who participated in the policy dialogue.

Stakeholder category	N = 24 (100%)
Policy-makers^a^	5 (20.8%)
Health-care providers^b^	11 (45.8%)
Researchers in the field of public and mental health^c^	6 (25%)
Civil society organization^d^	1 (4.2%)
Public defense representative^e^	1 (4.2%)

^a^Policy-makers at the federal, state, and municipal level; ^b^Health care providers included mental health specialists, public health specialists, psychologists, psychiatrists, occupational therapists, nurses, and social workers; ^c^Researchers from Brazilian public and private universities, EVIPNet-Brazil members, and Seriema members; ^d^The Brazilian anti-asylum movement; ^e^Public defense representative from the state of São Paulo who was involved in mental health-related legislations.

## Results

The results that are presented in the following sections summarise the main findings that pertain to the evidence brief; this section is followed by a discussion of the results that belong to the policy dialogue.

### Defining the Problem

*What are the most important challenges that impede the improvement of mental health care that is available to deinstitutionalized people with severe mental disorders in Brazil?*

The participants reviewed the findings that were presented in the evidence brief, highlighted what is already known about the problem, and provided an enriching analysis of the brief; this process consumed the most time. They individually and collectively focused on the prominent challenges: (i) insufficient and fragile community care services to meet patient needs; (ii) unequal access to community care across the different regions of Brazil; (iii) insufficient funding and a lack of political resources; (iv) qualitative and quantitative human resource deficiencies; (v) a lack of intensive follow-up care; and (vi) the absence of integration and communication between services.

All the participants agreed that it is necessary to expand and strengthen community care services for all Brazilians. Indeed, the process of deinstitutionalization did not progress in the same manner across different Brazilian regions. In some of them, such as region of Sorocaba (main manicomial pole), where the number of patients admitted to hospital beds was very high, the deinstitutionalization process exceeded the capacity of assimilation of services offered in community.

Some participants observed that, despite progress, community care services are still precarious with regard to a wide range of issues (i.e. from physical infrastructure to human resources). They noted that many professionals still retain an “asylum mentality,” and that there is insufficient communication among mental health professionals and services, and between the municipal, state, and federal governmental bodies. The participants contended that the lack of communication and continued education adversely affects the follow-up care and rehabilitation of patients with mental disorders.

The participants expressed their concerns about the process of deinstitutionalization (i.e. the withdrawal of patients from psychiatric hospitals) and trans-institutionalization (i.e. the transfer of patients from asylums to other inappropriate institutions). Indeed, these can lead to social neglect and have profound repercussions for the community, such as increased rates of homelessness, incarceration, drug addiction (primarily, cocaine), depression, suicide, and an overloading of emergency services. All participants agreed that deinstitutionalization requires efforts that extend beyond deinstitutionalization and trans-institutionalization. They also agreed that the “Ministry of Health must have a serious commitment to those patients who leave the psychiatric hospitals.”

The participants contended that the issue of deinstitutionalization is also complicated by financial conflicts of interests that pertain to psychiatric hospitalizations. This suggests that there is a “mercantilization of life of an especially vulnerable population.” Finally, the participants also recognised that the health care that is available to deinstitutionalized individuals has significantly advanced across the years; however, the socio-cultural treatment of these individuals remains problematic.

### Options to Address the Problem of Deinstitutionalization

The five mutually non-exclusive options to address the problem of deinstitutionalization that was articulated in the evidence brief are presented in **Table 2**.

**Table 2:** The definitions of the options to address deinstitutionalization that were presented in the evidence brief.

Option	Definition
**Option 1: Expand and Improve the Implementation of a Psychiatric Day Hospital**	It is a hospital unit that offers intensive care to patients with acute mental disorders based on a multidisciplinary approach and early discharge policy (Marshall et al., 2011).
**Option 2: Provide Psychoeducational programs**	Psychoeducation provides patients and their families or caregivers with information about the disease, its treatment, and its prognosis (Xia et al., 2011; Zhao et al., 2015).
**Option 3: Develop Community Mental Health Teams**	Multidisciplinary teams provide specialized mental health care to patients with mental disorders in the community, facilitate early intervention, and lower the rates of hospital admissions and suicides (Malone et al., 2007).
**Option 4: Implement and Monitor the Practice of Intensive Case Management**	It is a flexible model of mental health services that is characterised by intensive case management and patient care that is provided to individuals with mental disorders in the community. It is available throughout the day, and the follow-up care is provided by a multidisciplinary team to a small group of patients. They aim to improve social reintegration, psychosocial functioning, and autonomy development, and decrease the rate of hospitalization and treatment abandonment (Dieterich et al., 2010).
**Option 5: Promote assisted living**	Structuring housing intended to accommodate patients with mental disorders who have been hospitalized in psychiatric institutions for many years, and are currently homeless and unable to return to their families (Leff et al., 2009).

The deliberations that pertained to the options are summarised in the following sections.

#### Option 1: Expand and Improve the Implementation of a Psychiatric Hospital Day

This option caused much polemic and controversy among the participants of the policy dialogue, possibly because of a misunderstanding of the option. A majority of the participants opposed this option because they considered traditional psychiatric hospitals to be regressive: “*something that did not work in the past, which isolates and excludes*.” At the same time that the policy dialogue was conducted, the national policy on mental health was being reformulated with a strong aim to reopen the psychiatric hospitals; evidently, many of the participants were aware of this. However, other participants understood this option more accurately and were in favor of such an approach because it entails the early discharge policy of psychiatric day hospitals. However, they suggested that the name of the option be changed to “Strengthening interventions for acute psychiatric episodes” in order to convey that this option endorses institutions that provide humane treatment to individuals who present with acute psychiatric episodes, and hospitalize briefly such individuals only when necessary.

#### Option 2: Provide Psychoeducational Programs

This option was wholeheartedly supported and endorsed by the participants. Further, a majority of the studies that were reviewed supported the effectiveness of this option. The Brazilian Health System does not offer psychoeducational programs. According to some of the participants, this may have been attributable to the preconceived notions that managers hold about mental health professionals. They also recommended the implementation of a few psychoeducational techniques.

#### Option 3: Develop Community Mental Health Teams

Only one of the systematic reviews (Malone et al., 2007) addressed this option. Nevertheless, the conclusions of the review suggested that this option promotes greater acceptance of the treatment and greater patient satisfaction, when compared to standard treatment paradigms. In addition, the hospitalization rate was significantly lower; this suggests that the number of suicides and deaths under suspicious circumstances was also lower. The participants considered this option to be interesting and promising. However, the Brazilian mental health policy does not have provisions for such community mental health teams. Although Brazil does have other community teams, they comply with only a few of the principles of the proposed team.

#### Option 4: Implement and Monitor the Practice of Intensive Case Management

Model that is similar to those of intensive case management are practiced in some communities in Brazil. Every participant considered this model to be extremely important to all Brazilian cities. However, several small towns do not comply with this model. Therefore, the participants highlighted the importance of expanding and strengthening this model.

#### Option 5: Promote Assisted Living

The participants underscored the importance of and challenges that are involved in implementing assisted living in such a manner that it does not result in trans-institutionalization.

Two participants observed issues that pertained to the inadequacy of housing, infrastructure, and food, and the absence of leisure-time activities.

Many participants agreed that cohabitating a space with individuals who differ in age, diagnosis, and the severity of the diagnosis facilitates social reintegration: “caring and helping each other are positive factors observed in their daily lives.”

### The Evidence Brief and Policy Dialogue: Evaluation Results

Eight and nine individuals out of the 24 participants completed the evaluation surveys for the evidence brief and policy dialogue, respectively. The response rate was low despite repeated attempts to administer the survey, and it can be attributed to time limitations and the busy lives that the participants led.

Despite the low response rate, the average item scores were positive, and they ranged from 5.0 to 7.0 for the evidence brief evaluation survey. The features that received the highest ranking (i.e. very helpful) were as follows: employ a graded-entry format and use systematic and transparent methods to identify, select, and assess synthesized research evidence (see **Supplementary Materials—Table S2**).

The results of the policy dialogue evaluation were also positive, and the scores ranged from 4.6 to 6.6. The following features were considered to be very useful: rely on a facilitator to assist with the deliberation, address high-priority policy issues, do not aim for consensus, provide an exhaustive discussion, and ensure a fair representation of those who will be involved in or affected by future decisions that are related to the respective issue (see **Supplementary Materials—Table S3**).

### Post-Dialogue Mini-Interviews

Approximately 10 individuals agreed to participate in the post-dialogue mini-interviews, which were video-recorded. The findings of the study suggest that many participants demonstrated the positive insights that they gained during the policy dialogue (see **Table 3**).

**Table 3:** Participant opinions (insights) about the policy dialogue.

• “Very important space to discuss and align the thoughts so that the actions are more articulated”
• “Moment of interaction between different visions and access to information that goes well beyond global evidence … greatly influenced by different views and experiences”
• “It is extremely important that managers, members of civil society and academia come together to discuss mental health issues. Articulation between Ministry of Health, universities and various actors involved in mental health policy will contribute to the advancement of public health policies in mental health”
• “The opportunity to listen to people who work in different areas of mental health was very important to understand better the problem and to contextualize the policy brief developed”
• “Policy dialogue is very interesting because it is not a debate; people dialogue and reflect to evolve in a particular concept or a specific implementation policy … it allows the communication between the services of several levels”
• “Opportunity to bring together research and management … the research shows the theoretical component that management does not have”
• “An important approach between research and practice … does not seek a consensus, seeks a listening…”
• “It provides an expanded view of how deep the needs are around psychiatric reform in Brazil, and how divergent the opinions are from collecting local evidence from different actors in society (local, federal, professional, and civil society managers)…representing an environment of democratic discussion”
• “Listening to the most diverse opinions on the same subject, same problem … there are several actors involved and each one with a participation, experience and a point of view … very important this exchange, because it is very difficult to see from another prism”

## Discussion

### Main Findings

The application of KT tools to support efforts to improve the care of deinstitutionalized patients with mental disorders and to contribute to the promulgation of evidence-informed mental health policies was a promising and innovative experience in Sorocaba. This experience entailed eight steps, and it demonstrated to policy-makers that the process of KT can bridge the gap between research and practice.

The application of evidence in mental health practice and the exchange of knowledge between health-care providers, researchers, and community representatives were positively appraised. The entire process also helped those who are likely to be involved in or be affected by future policy-related decisions gain valuable insights. The features of the evidence brief and policy dialogue were considered to be very helpful, and they believed that it promoted an exhaustive discussion about the issue of deinstitutionalization.

### A Comparison of the Present and Past Findings

Capacity building, which was the first step of the process, made the participants aware of the importance of the following: using KT tools to make evidence-informed policy decisions, align research at the University of Sorocaba with policy priorities, and build partnerships between policy-makers, stakeholders, and researchers. Training workshops have been found to improve knowledge and comprehension about the use of evidence in policy decision making in other countries as well (Uneke et al., 2012; Waqa et al., 2013; El-Jardali et al., 2014). The workshops also strengthened partnerships and enhanced the interaction between the Seriema group and the Health Departments of Sorocaba and the neighboring regions.

The evidence brief was prepared based on the best evidence available on the issue at hand. However, a majority of the systematic reviews focused on high-income countries (e.g. the United States of America, the United Kingdom, Canada, Australia), and none of them were conducted in Brazil. This demonstrated a knowledge gap regarding mental health care in Brazil (Amaral et al., 2018; Votruba et al., 2018). This led to many difficulties because the relationship between evidence and policy-making depends on country-specific features (e.g. social, organizational, and public factors), the specific policy issue, resources allocation, and contextual factors, which are very different (and in some cases, deficient) in LMICs (Tricco et al., 2013; Votruba et al., 2018). This difference can be attributed to the following features that characterise LMICs: low research capacity, an obscure policy-making process, a high risk of political instability, limited financial resources, a lack of interaction between researchers and policy-makers, and lack of empowerment of civil society (Young, 2005).

Furthermore, our findings corroborate the gap between research and practice that has been observed in LMICs, as well as the difficulties and complexities that mental health care entails. Despite the global burden of mental disorders (e.g. disability and lower disability-adjusted life years), mental health is not a policy priority in LMICs (Patel, 2007; Votruba et al., 2018). Mental health policy issues differ from other policy issues because they pertain to a highly heterogeneous set of conditions (i.e. mental, behavioral, or neurodevelopmental disorders), the presence of comorbidities, a lack of consensus on the best possible approach to treatment and care, a high rate of untreated patients, and the incumbent stigma (Votruba et al., 2018).

The definition of the problem and the options were discussed exhaustively, without the aim of reaching a consensus. The problem was perceived to be critical, and many of the participants (policy-makers, health-care providers, researchers, and representative of civil society, and public defense) conceptualized the problem based on their rich practical experience, and they echoed a majority of the challenges that were already presented in the evidence brief. In other words, the policy dialogue deliberations validate the evidence brief (Yehia and El Jardali, 2015). Thus, it is noteworthy that option 2 (*Provide psychoeducational programs*) was strongly supported by findings as well as the participants. On the other hand, option 1 (*Expand and improve the implementation of a Psychiatric Day Hospital*) was strongly opposed by a majority of the participants due to local findings; further, there were differences of opinion between international and local researches. Many of the participants were aware of the grave and inhumane treatment that patients with mental disorders had been subjected to in psychiatric hospitals in this region; they were also cognisant of the struggles that were required to shut down all the hospitals. The regulation of care with regard to crisis management and the treatment of acute episodes appear to be the most unclear albeit critical aspects of mental health care in Brazil (Amaral et al., 2018).

There is no KT strategy that is singularly effective across all contexts. Therefore, it is important to report about the context-specific utility of each strategy, so that they can be modified and utilised by other interested decision makers (Larocca et al., 2012). In this study, the participants provided positive evaluations of the evidence brief and of policy dialogue; they considered it to be favorable and useful, and these results corroborate past findings (Yehia and El Jardali, 2015; Boyko et al., 2016; Mc Sween-Cadieux et al., 2018). Similar findings emerged from the mini-interviews that were conducted at the end of the policy dialogue; specifically, all participant opinions were positive in tone. The use of a facilitator be to assist with the deliberation was considerate the most helpful feature of the policy dialogue. Past findings corroborate these results and emphasize the role of the facilitator as an unbiased agent which support KT platform (El-Jardali et al., 2014; Yehia and El Jardali, 2015).

Evidence briefs and summaries of policy dialogues (i.e. products of KT) can be used in public health policy-making only if the local and federal authorities are receptive to such efforts; unfortunately, often not the case (Cabieses and Espinoza, 2011).

Although the application of KT in public health policy-making is relatively new in LMCIs, the situation is changing. There is an increased use of evidence-informed policy frameworks (Cabieses and Espinoza, 2011; Votruba et al., 2018) and an increased demand for KT products from policy-makers. This has been proven by the EVIPNet-Brazil, which has expanded and consolidated its network (Dias et al., 2014). This practice needs to become a priority for Brazilian policy-makers because evidence-based public health models are powerful frameworks that can be used to identify the most effective health strategies and ensure that the resources are spent appropriately (Milat and Li, 2017).

### Limitations and Strengths

The present study was the first attempt to use KT tools to improve some aspects of mental health care in Brazil (e.g. deinstitutionalization), which is a priority topic of regional and national importance. The policy dialogue brought together stakeholders who are involved in the process of deinstitutionalization (e.g. researchers, policy-makers, health-care providers, and representatives from public defense and civil society), which enriched the deliberations and provided the participants with an opportunity to acquire new knowledge and learn from each other.

The present study has a few limitations. A large part of the KT framework and the best evidence available were developed in high-income countries (e.g. the United Kingdom, Canada, Australia) that’s can bring indirectness evidence. Further, we could not examine budgetary impact because the studies did not present cost analyses. Additionally, some of the options that were identified were difficult to understand because they were articulated using obscure terminologies. The variability in the quality of the reviewed studies and the lack of information about the options that can be implemented are a few other limitations. The low response rate that was evidenced for the evidence brief and policy dialogue evaluation surveys was attributed to time limitations and the busy lives that our participants led; therefore, some of our results may be underestimated. Although we have conducted an exhaustive and in-depth discussion, some topics that pertained to implementation were not discussed due to the paucity of time. However, since some aspects of implementation vary across communities, they should be discussed in accordance with the conditions of each municipality.

## Conclusions

The KT process that was adopted was considered to be a useful means to discuss important policy issues, bring together policy-makers, health care providers, researchers, and representatives of civil society and public defense, enhance interaction and partnerships between evidence-producers and evidence-users, and promote the dissemination and application of global and local evidence in practice.

The present study did not seek to examine causal relationships. Nevertheless, a longer study period will allow future researchers to capture the positive changes in mental health care that result from KT. Future investigations are required to understand whether and how evidence briefs and policy dialogue can be used to improve the care of deinstitutionalized people with severe mental disorders and their contributions to Brazilian mental health policy.

Researchers and other stakeholders who are interested in using KT tools should consider the lessons that were learnt during the course of our study.

## Author Contributions

LL conceptualized the study. IF, JB, LL designed the study. IF, SB, MC and LL led data collection, carried out the analysis and drafted the initial manuscript. All authors read (IF, LL, JB, SB and MC) provided critical revision and approved the final manuscript.

## Funding

This article was funded by FAPESP grants 2017/20668-7 and EVIPNet Brazil/Ministry of Health SCON2017-02502.

## Conflict of Interest

The authors declare that the research was conducted in the absence of any commercial or financial relationships that could be construed as a potential conflict of interest.
