# Supplementary material for: Knowledge Translation for Improving the Care of Deinstitutionalized People With Severe Mental Illness in Health Policy
Source: Front Pharmacol. 2020 Jan 21;10:1470. doi: 10.3389/fphar.2019.01470 (PMC6985550; doi:10.3389/fphar.2019.01470)
Supplement: Supplementary file 1 [file DataSheet_1.pdf]

Systematic Search for the development of an evidence brief to address the problem of deinstitutionalisation

Supplementary material - Data Sheet 1

Once the issue of deinstitutionalisation was prioritised, the focus was geared towards gathering a wide range of evidence relevant on the various aspects of the issue. Firstly, a systematic search was conducted using the following research databases: *Virtual Health Library, The Cochrane Library, PubMed, Health Evidence, Rx for Change, The Cumulative Index to Nursing and Allied Health Literature (CINAHL), Excerpta Medica Database (EMBASE), American Psychological Association (PsycINFO), Epistemonikos, Latin American & Caribbean Health Sciences Literature (LILACS), the Health System Performance Index (IDSUS), the Strategic Management Support Room (SAGE), and the National Collection of Health Resources of the Department of Informatics of the Brazilian Unified Health System (COLECIONASUS).* We used a well-defined search strategy, and we did not place restrictions based on the language or date of publication.

The retrieval of articles was conducted using a combination of the following terms: *'Deinstitutionalization', 'Mental Disorders', 'Community Mental Health Services', 'Case Management', 'Managed Care Programs', 'Community Mental Health Centres', 'Supported Housing', 'Psychoeducation', 'Community Mental Health Team', 'Crisis Intervention'.* These terms were used irrespective of whether they were indexed in the Medical Subject Headings (MeSH) System. We also manually searched the reference lists and citations of secondary studies to identify eligible studies.

Subsequently, we selected articles that pertained to the policies that are related to the care of deinstitutionalised patients with mental disorders. The quality of the systematic reviews was assessed using the measurement tool for the 'Assessing the Methodological Quality of Systematic Reviews'(AMSTAR).
