# Supplementary material for: Knowledge Translation for Improving the Care of Deinstitutionalized People With Severe Mental Illness in Health Policy
Source: Front Pharmacol. 2020 Jan 21;10:1470. doi: 10.3389/fphar.2019.01470 (PMC6985550; doi:10.3389/fphar.2019.01470)
Supplement: Supplementary file 2 [file Table_1.docx]

**THE IDENTIFICATION AND PRIORITIZATION OF THEMES**

***(Supplementary Material – Table S1)***

**Table S1: Specify, in a few words, the problem that you would like to work with SERIEMA group:**

| **About your initial perception of the problem** |  | **Comments** |
| --- | --- | --- |
| a) Is it a prevalent health problem in the population? | ○Yes  ○ No |  |
| b) Does the problem jeopardize service delivery or local health policy? | ○ Yes  ○ No |  |
| c) Are there local policies or guidelines on the problem? | ○ Yes  ○ No |  |
| c) Are there local policies or guidelines on the problem? | ○ Yes  ○ No |  |
| e) Does the problem induce unnecessary costs to the Brazilian Health System? | ○ Yes  ○ No |  |
| f) Are the team and decision makers willing to solve the problem? | ○ Yes  ○ No |  |

We will select criteria to prioritize the problems to be addressed within SERIEMA group. We will use the form below to identify the most important criteria.

Consider the scale below:

- **1 points out the unimportant criteria;**
- **5 indicate the most important criteria.**
- **Then evaluate how the problem highlighted above relates to the corresponding criteria.**
- **In this case, the scale will start at 1 (no impact of the problem on the criteria) up to 5 (high impact of the problem on the criteria).**

| **Criteria** | **Criteria weight** | **Impact of the problem** |
| --- | --- | --- |
| 1. Severity and prevalence of health condition | ○ 1 ○ 2 ○ 3 ○ 4 ○ 5 | ○ 1 ○ 2 ○ 3 ○ 4 ○ 5 |
| 2. Social cost of health status | ○ 1 ○ 2 ○ 3 ○ 4 ○ 5 | ○ 1 ○ 2 ○ 3 ○ 4 ○ 5 |
| 3. Potential results of evidence synthesis to improve outcome/benefit to health | ○ 1 ○ 2 ○ 3 ○ 4 ○ 5 | ○ 1 ○ 2 ○ 3 ○ 4 ○ 5 |
| 4. Potential results of the synthesis of evidence to change costs for the health system | ○ 1 ○ 2 ○ 3 ○ 4 ○ 5 | ○ 1 ○ 2 ○ 3 ○ 4 ○ 5 |
| 5. Potential results of the synthesis of evidence to contribute to the improvement of quality of care | ○ 1 ○ 2 ○ 3 ○ 4 ○ 5 | ○ 1 ○ 2 ○ 3 ○ 4 ○ 5 |
| 6. Potential results of evidence synthesis in reducing health risks | ○ 1 ○ 2 ○ 3 ○ 4 ○ 5 | ○ 1 ○ 2 ○ 3 ○ 4 ○ 5 |
| 7. Unit or aggregate cost of the problem | ○ 1 ○ 2 ○ 3 ○ 4 ○ 5 | ○ 1 ○ 2 ○ 3 ○ 4 ○ 5 |
| 8. Sufficient availability of scientific evidence | ○ 1 ○ 2 ○ 3 ○ 4 ○ 5 | ○ 1 ○ 2 ○ 3 ○ 4 ○ 5 |
| 9. Controversy or great interest among health professionals | ○ 1 ○ 2 ○ 3 ○ 4 ○ 5 | ○ 1 ○ 2 ○ 3 ○ 4 ○ 5 |
| 10. Requirements of State actions | ○ 1 ○ 2 ○ 3 ○ 4 ○ 5 | ○ 1 ○ 2 ○ 3 ○ 4 ○ 5 |

Please register your name and email to resolve any questions:

Specify, in a few words, another problem that you would like to work with SERIEMA group:

|  |
| --- |
