# Supplementary material for: Knowledge Translation for Improving the Care of Deinstitutionalized People With Severe Mental Illness in Health Policy
Source: Front Pharmacol. 2020 Jan 21;10:1470. doi: 10.3389/fphar.2019.01470 (PMC6985550; doi:10.3389/fphar.2019.01470)
Supplement: Supplementary file 3 [file Table_2.docx]

**The evidence brief evaluation survey items**

***(Supplementary Material – Table S2)***

**Table S2: The evidence brief evaluation survey items.**

| **Questions pertaining to design features^a^** | **Mean^b^**  **n=8** |
| --- | --- |
| Described the relevant context and different characteristics of the problem | 6.5 |
| Described some options to address the problem | 6.5 |
| Described key implementation considerations of the options | 6.5 |
| Quality considerations when discussing the research evidence | 6.5 |
| Local applicability considerations when discussing the research evidence | 5.0 |
| Equity considerations into when discussing the research evidence | 6.0 |
| Evidence brief employed a graded-entry format | 7.0 |
| Evidence brief reviewed for both scientific quality and system relevance | 6.0 |
| Evidence brief employed systematic and transparent methods to identify, select and assess synthesised research evidence | 7.0 |

^a^The questions were adapted from Lavis et al^8^.

^b^The response scale ranged from 1 (very unhelpful) to 7 (very helpful).
