# Supplementary material for: Knowledge Translation for Improving the Care of Deinstitutionalized People With Severe Mental Illness in Health Policy
Source: Front Pharmacol. 2020 Jan 21;10:1470. doi: 10.3389/fphar.2019.01470 (PMC6985550; doi:10.3389/fphar.2019.01470)
Supplement: Supplementary file 4 [file Table_3.docx]

**The policy dialogue evaluation survey items**

***(Supplementary Materials – Table S3)***

**Table: The policy dialogue evaluation survey items.**

| **Questions pertaining to design features^a^** | **Mean^b^**  **n=9** |
| --- | --- |
| Addressed high-priority policy issues | 6.6 |
| Provided an opportunity to discuss different aspects of the issues | 6.4 |
| Provided an opportunity to discuss possible options for addressing issues | 5.7 |
| Provided an opportunity to discuss key implementation considerations | 4.7 |
| Provided an opportunity to discuss who might do what differently | 5.8 |
| Deliberative was informed by a pre-circulated issue brief | 6.1 |
| Included discussion about factors that can inform how to approach the issues, possible options for addressing them and key implementation considerations | 6.3 |
| Ensured fair representation among those who will be involved or affected by future decisions related to the issue | 6.5 |
| Engaged a facilitator to assist with the deliberation | 6.7 |
| Allowed for frank, off-the-record deliberations following the Chatham House Rule | 6.2 |
| Did not aim for consensus | 6.4 |
| Reached the goal of promoting an exhaustive discussion | 6.5 |

^a^ The questions were adapted from Lavis et al.^8^ and Boyko et al^37^.

^b^ The response scale ranged from 1 (very unhelpful) to 7 (very helpful).
